# 4-(1*H*-Pyrazol-3-yl)pyridine–terephthalic acid–water (2/1/2)

**DOI:** 10.1107/S1600536812021599

**Published:** 2012-05-31

**Authors:** Zheng-De Tan, Feng-Jiao Tan, Bo Tan, Cheng-Ming Zhang

**Affiliations:** a College of Chemistry and Chemical Engineering, Hunan Institute of Engineering, Xiang Tan 411104, People’s Republic of China; b The People’s Hospital of Xiangtan County, Xiang Tan 411104, People’s Republic of China

## Abstract

In the title compound, 2C_8_H_7_N_3_·C_8_H_6_O_4_·2H_2_O, the pyridine and pyrazole rings are approximately coplanar, the dihedral angle between them being 4.69 (9)°. The asymmetric unit consists of half of the terephthalic acid (an inversion centre generates the other half of the mol­ecule), one 4-(1*H*-pyrazol-3-yl)pyridine (4pp) mol­ecule and one water mol­ecule. In the crystal, two 4pp and one terephthalic acid mol­ecules form a linear three-molecule unit as a result of O—H⋯N hydrogen bonds. These units are further assembled into a three-dimensional network by two types of hydrogen bonds, *viz.* O—H⋯O and N—H⋯O.

## Related literature

For the synthesis of 4-(1*H*-pyrazol-3-yl)-pyridine, see: Davies *et al.* (2003 ▶).
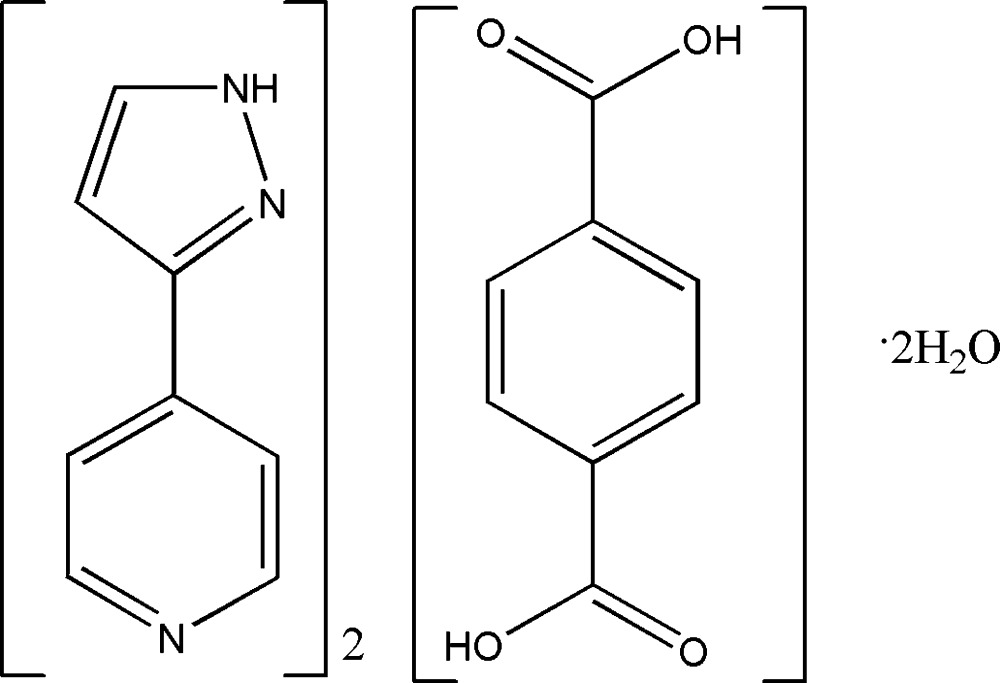


## Experimental

### 

#### Crystal data

2C_8_H_7_N_3_·C_8_H_6_O_4_·2H_2_O
*M* *_r_* = 492.49
Triclinic, 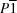

*a* = 6.8364 (14) Å
*b* = 9.5308 (19) Å
*c* = 10.131 (2) Å
α = 67.52 (3)°
β = 71.22 (3)°
γ = 78.10 (3)°
*V* = 574.9 (2) Å^3^
*Z* = 1
Mo *K*α radiation
μ = 0.11 mm^−1^
*T* = 293 K
0.32 × 0.25 × 0.18 mm

#### Data collection

Rigaku SCXmini diffractometer
Absorption correction: multi-scan (*ABSCOR*; Higashi, 1995 ▶) *T*_min_ = 0.967, *T*_max_ = 0.981
5041 measured reflections
2024 independent reflections
1254 reflections with *I* > 2σ(*I*)
*R*_int_ = 0.054

#### Refinement

*R*[*F* ^2^ > 2σ(*F* ^2^)] = 0.064
*wR*(*F* ^2^) = 0.128
*S* = 1.21
2024 reflections
172 parameters
4 restraints
H atoms treated by a mixture of independent and constrained refinement
Δρ_max_ = 0.32 e Å^−3^
Δρ_min_ = −0.28 e Å^−3^

### 

Data collection: *PROCESS-AUTO* (Rigaku, 1998 ▶); cell refinement: *PROCESS-AUTO*; data reduction: *PROCESS-AUTO*; program(s) used to solve structure: *SHELXS97* (Sheldrick, 2008 ▶); program(s) used to refine structure: *SHELXL97* (Sheldrick, 2008 ▶); molecular graphics: *DIAMOND* (Brandenburg & Putz, 2005 ▶); software used to prepare material for publication: *SHELXL97*.

## Supplementary Material

Crystal structure: contains datablock(s) I, global. DOI: 10.1107/S1600536812021599/nk2153sup1.cif

Structure factors: contains datablock(s) I. DOI: 10.1107/S1600536812021599/nk2153Isup2.hkl

Additional supplementary materials:  crystallographic information; 3D view; checkCIF report

## Figures and Tables

**Table 1:** Hydrogen-bond geometry (Å, °)

*D*—H⋯*A*	*D*—H	H⋯*A*	*D*⋯*A*	*D*—H⋯*A*
N1—H1⋯O1*W*^i^	0.86	1.98	2.829 (3)	170
O1*W*—H1*W*⋯O2^ii^	0.84 (1)	1.99 (1)	2.811 (3)	167 (2)
O1*W*—H2*W*⋯O1^iii^	0.84 (1)	2.06 (1)	2.864 (3)	161 (2)
O1—H11⋯N3	0.82 (1)	1.80 (1)	2.614 (3)	170 (3)

Symmetry codes: (i) 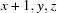; (ii) 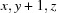; (iii) 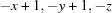.

**Fig. 1.**
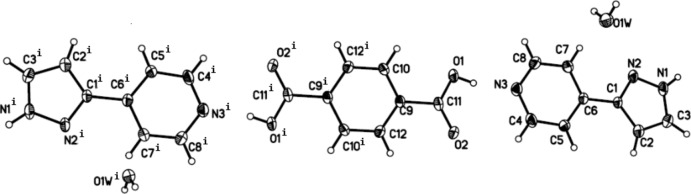
The structure of the title compound, with 30% probability displacement ellipsoids [Symmetry codes: i = -x, -y, -z].

**Fig. 2.**
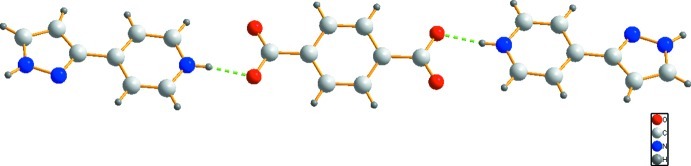
A view of the supermolecule unit of the title compound. Hydrogen bonds are shown as dashed lines.

**Fig. 3.**
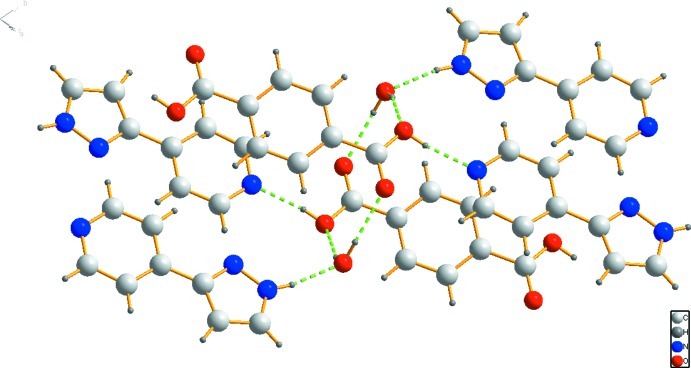
Three types hydrogen bonds in the stucture. Hydrogen bonds are shown as dashed lines.

## supplementary crystallographic information

### Comment

In the title compound, the pyridine ring and the pyrazole ring are approximately coplanar with the dihedral angles between them being 4.69 (9)°. Two 4pp and one terephthalic acid form a linear three-molecule unit as a result of O—H···N hydrogen bonds (Fig.2 and Table 1), which the N atom is from the ring of pyridine.There is a hydrogen interaction between N1 from pyrazol as the hydrogen bond donor and O1w as the hydrogen bond acceptor.At the same time, O1w as the hydrogen bond donor interacts with two O2 atoms from different terephthalic acid (Fig.3). These supermolecules are assembled into a three-dimensional network by two types of hydrogen bonding including O—H···O and N—H···O.

### Experimental

4-(1*H*-pyrazol-3-yl)-pyridine was prepared according to the published method of Davies *et al.* (2003). An aqueous solution (20 mL) containing terephthalic acid( 0.1 mmol,16 mg), NaOH (0.2 mmol,8 mg) and 4-(1*H*-pyrazol-3-yl)-pyridine (0.2 mmol,29 mg) was stirred for 20 minutes in air, and left to stand at room temperature for about four weeks, then the colorless crystals were obtained.

### Refinement

C- and N- bound H atoms were placed at calculated positions and were treated as riding on the parent C or N atoms with C—H = 0.93 Å, N—H = 0.86 Å, and with *U*_iso_(H) = 1.2 *U*_eq_(C, N). The water H-atoms were located in a difference map, and were refined with a distance restraint of O—H = 0.84 Å; their *U*_iso_ values were refined.

### Crystal data

**Table:**

2C_8_H_7_N_3_·C_8_H_6_O_4_·2H_2_O	*Z* = 1
*M**_r_* = 492.49	*F*(000) = 258
Triclinic, *P*1	*D*_x_ = 1.423 Mg m^−^^3^
Hall symbol: -P 1	Mo *K*α radiation, λ = 0.71073 Å
*a* = 6.8364 (14) Å	Cell parameters from 4645 reflections
*b* = 9.5308 (19) Å	θ = 3.2–27.5°
*c* = 10.131 (2) Å	µ = 0.11 mm^−^^1^
α = 67.52 (3)°	*T* = 293 K
β = 71.22 (3)°	Block, colourless
γ = 78.10 (3)°	0.32 × 0.25 × 0.18 mm
*V* = 574.9 (2) Å^3^	

### Data collection

**Table:**

Rigaku SCXmini diffractometer	2024 independent reflections
Radiation source: sealed tube	1254 reflections with *I* > 2σ(*I*)
Graphite monochromator	*R*_int_ = 0.054
ω scans	θ_max_ = 25.0°, θ_min_ = 3.2°
Absorption correction: multi-scan (*ABSCOR*; Higashi, 1995)	*h* = −8→8
*T*_min_ = 0.967, *T*_max_ = 0.981	*k* = −11→11
5041 measured reflections	*l* = −12→12

### Refinement

**Table:**

Refinement on *F*^2^	Primary atom site location: structure-invariant direct methods
Least-squares matrix: full	Secondary atom site location: difference Fourier map
*R*[*F*^2^ > 2σ(*F*^2^)] = 0.064	Hydrogen site location: inferred from neighbouring sites
*wR*(*F*^2^) = 0.128	H atoms treated by a mixture of independent and constrained refinement
*S* = 1.21	*w* = 1/[σ^2^(*F*_o_^2^) + (0.0423*P*)^2^] where *P* = (*F*_o_^2^ + 2*F*_c_^2^)/3
2024 reflections	(Δ/σ)_max_ = 0.002
172 parameters	Δρ_max_ = 0.32 e Å^−^^3^
4 restraints	Δρ_min_ = −0.28 e Å^−^^3^

### Special details

**Table:**

Geometry. All e.s.d.'s (except the e.s.d. in the dihedral angle between two l.s. planes) are estimated using the full covariance matrix. The cell e.s.d.'s are taken into account individually in the estimation of e.s.d.'s in distances, angles and torsion angles; correlations between e.s.d.'s in cell parameters are only used when they are defined by crystal symmetry. An approximate (isotropic) treatment of cell e.s.d.'s is used for estimating e.s.d.'s involving l.s. planes.
Refinement. Refinement of *F*^2^ against ALL reflections. The weighted *R*-factor *wR* and goodness of fit *S* are based on *F*^2^, conventional *R*-factors *R* are based on *F*, with *F* set to zero for negative *F*^2^. The threshold expression of *F*^2^ > σ(*F*^2^) is used only for calculating *R*-factors(gt) *etc*. and is not relevant to the choice of reflections for refinement. *R*-factors based on *F*^2^ are statistically about twice as large as those based on *F*, and *R*- factors based on ALL data will be even larger.

### Fractional atomic coordinates and isotropic or equivalent isotropic displacement parameters (Å^2^)

**Table:**

	*x*	*y*	*z*	*U*_iso_*/*U*_eq_	
C1	1.0188 (4)	0.3344 (3)	0.3605 (3)	0.0315 (7)	
H1W	0.422 (4)	0.7787 (14)	0.275 (3)	0.047*	
N1	1.2378 (3)	0.4525 (3)	0.3732 (3)	0.0425 (7)	
H1	1.3209	0.5197	0.3507	0.051*	
O1	0.3502 (3)	0.2110 (2)	0.0458 (2)	0.0473 (6)	
O1W	0.4882 (3)	0.6952 (2)	0.2700 (2)	0.0513 (6)	
C2	1.0696 (4)	0.2524 (3)	0.4943 (3)	0.0420 (8)	
H2	1.0189	0.1622	0.5651	0.050*	
H2W	0.553 (4)	0.702 (3)	0.1821 (10)	0.063*	
N2	1.1240 (4)	0.4575 (3)	0.2854 (2)	0.0400 (7)	
O2	0.3105 (3)	−0.0044 (2)	0.2397 (2)	0.0475 (6)	
C3	1.2093 (5)	0.3327 (4)	0.4991 (3)	0.0444 (8)	
H3	1.2723	0.3089	0.5749	0.053*	
N3	0.6010 (3)	0.2457 (3)	0.1765 (3)	0.0396 (6)	
C4	0.6356 (4)	0.1461 (3)	0.3035 (3)	0.0449 (8)	
H4	0.5675	0.0577	0.3498	0.054*	
C5	0.7685 (4)	0.1702 (3)	0.3678 (3)	0.0418 (8)	
H5	0.7896	0.0987	0.4563	0.050*	
C6	0.8716 (4)	0.3018 (3)	0.3005 (3)	0.0311 (7)	
C7	0.8296 (4)	0.4054 (3)	0.1688 (3)	0.0421 (8)	
H7	0.8919	0.4962	0.1210	0.050*	
C8	0.6973 (4)	0.3730 (3)	0.1105 (3)	0.0451 (8)	
H8	0.6734	0.4420	0.0218	0.054*	
C9	0.1342 (4)	0.0404 (3)	0.0568 (3)	0.0303 (7)	
C10	0.0548 (4)	0.1468 (3)	−0.0570 (3)	0.0362 (7)	
H10	0.0913	0.2463	−0.0964	0.043*	
C11	0.2749 (4)	0.0835 (3)	0.1202 (3)	0.0365 (7)	
H11	0.427 (3)	0.212 (3)	0.093 (2)	0.044*	
C12	0.0774 (4)	−0.1067 (3)	0.1123 (3)	0.0368 (7)	
H12	0.1291	−0.1799	0.1882	0.044*	

### Atomic displacement parameters (Å^2^)

**Table:**

	*U*^11^	*U*^22^	*U*^33^	*U*^12^	*U*^13^	*U*^23^
C1	0.0348 (17)	0.0340 (17)	0.0324 (17)	−0.0076 (14)	−0.0138 (14)	−0.0124 (15)
N1	0.0410 (16)	0.0508 (17)	0.0497 (17)	−0.0133 (12)	−0.0191 (13)	−0.0218 (15)
O1	0.0540 (14)	0.0522 (14)	0.0513 (14)	−0.0250 (11)	−0.0301 (11)	−0.0111 (12)
O1W	0.0522 (16)	0.0496 (14)	0.0540 (14)	−0.0137 (11)	−0.0216 (12)	−0.0092 (12)
C2	0.0446 (19)	0.0435 (19)	0.0449 (19)	−0.0102 (15)	−0.0174 (16)	−0.0152 (16)
N2	0.0418 (15)	0.0459 (16)	0.0419 (15)	−0.0168 (12)	−0.0179 (12)	−0.0134 (13)
O2	0.0631 (15)	0.0476 (14)	0.0426 (13)	−0.0174 (11)	−0.0317 (12)	−0.0065 (12)
C3	0.050 (2)	0.050 (2)	0.043 (2)	−0.0045 (16)	−0.0243 (16)	−0.0161 (18)
N3	0.0368 (15)	0.0468 (16)	0.0402 (16)	−0.0107 (12)	−0.0117 (12)	−0.0156 (14)
C4	0.045 (2)	0.049 (2)	0.048 (2)	−0.0232 (16)	−0.0101 (16)	−0.0169 (18)
C5	0.0507 (19)	0.0396 (18)	0.0389 (18)	−0.0182 (15)	−0.0195 (15)	−0.0043 (15)
C6	0.0322 (17)	0.0341 (17)	0.0327 (17)	−0.0028 (14)	−0.0119 (14)	−0.0149 (15)
C7	0.0468 (19)	0.0375 (18)	0.048 (2)	−0.0141 (14)	−0.0211 (16)	−0.0090 (16)
C8	0.049 (2)	0.048 (2)	0.0427 (19)	−0.0108 (17)	−0.0227 (16)	−0.0090 (17)
C9	0.0263 (16)	0.0353 (18)	0.0329 (16)	−0.0078 (13)	−0.0074 (13)	−0.0134 (15)
C10	0.0382 (18)	0.0311 (16)	0.0435 (18)	−0.0124 (14)	−0.0149 (15)	−0.0094 (15)
C11	0.0349 (17)	0.0402 (19)	0.0439 (19)	−0.0119 (15)	−0.0123 (15)	−0.0190 (17)
C12	0.0381 (18)	0.0401 (18)	0.0364 (17)	−0.0093 (14)	−0.0177 (14)	−0.0081 (15)

### Geometric parameters (Å, º)

**Table:**

C1—N2	1.336 (3)	C4—C5	1.373 (4)
C1—C2	1.397 (4)	C4—H4	0.9300
C1—C6	1.465 (3)	C5—C6	1.391 (3)
N1—C3	1.337 (3)	C5—H5	0.9300
N1—N2	1.340 (3)	C6—C7	1.400 (4)
N1—H1	0.8600	C7—C8	1.364 (4)
O1—C11	1.272 (3)	C7—H7	0.9300
O1—H11	0.8202 (11)	C8—H8	0.9300
O1W—H1W	0.8400 (11)	C9—C12	1.383 (3)
O1W—H2W	0.8400 (11)	C9—C10	1.391 (4)
C2—C3	1.364 (4)	C9—C11	1.506 (4)
C2—H2	0.9300	C10—C12^i^	1.382 (4)
O2—C11	1.247 (3)	C10—H10	0.9300
C3—H3	0.9300	C12—C10^i^	1.382 (4)
N3—C8	1.333 (3)	C12—H12	0.9300
N3—C4	1.335 (4)		
			
N2—C1—C2	110.9 (2)	C5—C6—C7	116.8 (3)
N2—C1—C6	120.5 (2)	C5—C6—C1	123.3 (2)
C2—C1—C6	128.6 (3)	C7—C6—C1	120.0 (2)
C3—N1—N2	113.3 (2)	C8—C7—C6	120.0 (3)
C3—N1—H1	123.3	C8—C7—H7	120.0
N2—N1—H1	123.3	C6—C7—H7	120.0
C11—O1—H11	102.3 (19)	N3—C8—C7	122.2 (3)
H1W—O1W—H2W	111.1 (12)	N3—C8—H8	118.9
C3—C2—C1	105.4 (3)	C7—C8—H8	118.9
C3—C2—H2	127.3	C12—C9—C10	118.0 (2)
C1—C2—H2	127.3	C12—C9—C11	120.6 (3)
C1—N2—N1	104.1 (2)	C10—C9—C11	121.4 (2)
N1—C3—C2	106.3 (3)	C12^i^—C10—C9	120.9 (3)
N1—C3—H3	126.8	C12^i^—C10—H10	119.5
C2—C3—H3	126.8	C9—C10—H10	119.5
C8—N3—C4	119.1 (2)	O2—C11—O1	124.0 (3)
N3—C4—C5	122.0 (3)	O2—C11—C9	119.7 (3)
N3—C4—H4	119.0	O1—C11—C9	116.3 (3)
C5—C4—H4	119.0	C9—C12—C10^i^	121.1 (3)
C4—C5—C6	120.0 (3)	C9—C12—H12	119.4
C4—C5—H5	120.0	C10^i^—C12—H12	119.4
C6—C5—H5	120.0		

Symmetry code: (i) −*x*, −*y*, −*z*.

### Hydrogen-bond geometry (Å, º)

**Table:**

*D*—H···*A*	*D*—H	H···*A*	*D*···*A*	*D*—H···*A*
N1—H1···O1*W*^ii^	0.86	1.98	2.829 (3)	170
O1*W*—H1*W*···O2^iii^	0.84 (1)	1.99 (1)	2.811 (3)	167 (2)
O1*W*—H2*W*···O1^iv^	0.84 (1)	2.06 (1)	2.864 (3)	161 (2)
O1—H11···N3	0.82 (1)	1.80 (1)	2.614 (3)	170 (3)

Symmetry codes: (ii) *x*+1, *y*, *z*; (iii) *x*, *y*+1, *z*; (iv) −*x*+1, −*y*+1, −*z*.
